# Health system challenges affecting falls prevention in persons living with HIV: perspectives from physiotherapists in four sub-Saharan regions

**DOI:** 10.1017/S1463423620000663

**Published:** 2021-09-13

**Authors:** Maria Y. Charumbira, Karina Berner, Quinette A. Louw

**Affiliations:** Division of Physiotherapy, Department of Health and Rehabilitation Sciences, Faculty of Medicine and Health Sciences, Stellenbosch University, Cape town, South Africa

**Keywords:** Falls, Health system, HIV, sub-Saharan Africa

## Abstract

**Aim:**

The aim of this study was to explore the perspectives of physiotherapists in four selected regions of sub-Saharan Africa regarding health system challenges impacting the integration of physiotherapy-led falls prevention services in the primary care of persons living with HIV (PLWH).

**Background:**

Falls may pose a significant problem among younger PLWH in low- and middle-income countries. Physiotherapists’ role in optimising function and quality of life can do much in the prevention of falls in PLWH and reducing the harm that results. However, falls prevention strategies have not been implemented effectively especially in primary health care settings in sub-Saharan Africa. Physiotherapists’ account of the health system challenges they encounter may provide insights into potential strategies that may be considered in optimising fall prevention for PLWH in poorly resourced settings.

**Methods:**

A descriptive qualitative study was conducted in selected urban districts in the capital cities of four sub-Saharan African countries. In-depth interviews were conducted with 21 purposively selected physiotherapists involved in the primary care of PLWH. Audio recordings of interviews were transcribed verbatim and analysed using deductive thematic content analysis.

**Findings:**

The main results are presented in the theme ‘Health care system challenges’ and in nine categories informed by the WHO health system framework: lack of policies and clinical practice guidelines, shortage/Inaccessible falls prevention services, inadequate human resource, physiotherapists not adequately equipped in falls prevention, inaccessible/No facilities for BMD measurement, inefficient data capturing systems, lack of evidence regarding falls among PLWH, unclear physiotherapy role descriptions, inefficient referral system. Physiotherapists highlighted the need for more information and research regarding fall prevention for PLWH, promote their role in the primary care of PLWH and adopt a patient-centred approach to fall prevention.

## Background

Falls are of increasing concern among persons living with HIV (PLWH) subsequent to improved access to more potent regimens of combination antiretroviral therapy (cART) (Erlandson *et al.*, 2012). The problem may be more profound in sub-Saharan Africa, where the biggest HIV epidemic in the world exists (GBD 2017 Disease and Injury Incidence and Prevalence Collaborators, 2018). Eastern and Southern Africa alone accounted for 53% of the global burden of HIV infection in 2017 (UNAIDS, 2018). Increased longevity and increased HIV prevalence resulting from improved ART access implies that more PLWH are ageing with the condition, while fall rates similar to older adults in the general population have been reported in middle-aged PLWH (Ruiz *et al.*, 2013). Studies from high-income countries (HICs) report a fall prevalence in middle-aged and older PLWH ranging from 12 to 41% (Richert *et al.*, 2014; Erlandson *et al.*, 2019); while preliminary findings from a recent South African study reported a prevalence of 34% among a relatively younger cohort of PLWH (median age = 36.61 years) (Berner *et al.*, 2019). Reduction in bone mineral density (BMD) related to exposure to antiretroviral drugs as well as viral infection of osteoblasts have been observed in PLWH (Yin and Falutz, 2017). As a quantitative measure of bone mass, BMD refers to the amount of calcium in grams per square centimetre of bone and clinically serves as a surrogate marker of osteoporosis and fracture risk (Kruger and Nell, 2017). The coexistence of falls and reduced BMD in PLWH further compounds the risk of fractures (Hoy and Young, 2016); resulting in functional decline, hospitalisation, institutionalisation, disability and even death (Premaor and Compston, 2018). Additionally, unresolved ART-induced neurotoxicity and virologic inflammation may impact negatively on the mobility of PLWH at younger-than-expected ages, in turn affecting their quality of life (QoL) (Greene, Justice and Covinsky, 2017).

The risk factors for falls in PLWH are largely modifiable (Erlandson *et al.*, 2012). Physiotherapists’ role in optimising function and QoL can do much in the primary and secondary prevention of falls in PLWH and reducing the harm that results (Dizon *et al.*, 2016). Screening and addressing variables of physical function such as balance impairments and muscle weakness may effectively manage challenges arising from reduced BMD, falls and fall-related fractures (Veeravelli *et al.*, 2016; Perazzo *et al.*, 2018). However, primary care is an emerging area for rehabilitation specialists (Phelan *et al.*, 2015; Mackenzie and Clifford, 2018). Physiotherapists’ efforts to implement fall prevention strategies at primary care level have been met with several barriers, some embedded within the health system (Liddle *et al.*, 2018).

Research to date has identified health system challenges affecting the implementation of falls prevention programmes for older adults in the general population (Child *et al.*, 2012). These included financial costs, low coverage or reimbursement by medical insurance schemes, inadequate and undertrained staff and lack of patient involvement in their treatment (Child *et al.*, 2012). Persons living with HIV are particularly challenged with stigma, multimorbidity, episodic disability, loss of social support and financial constraints (Cobbing, Hanass-Hancock and Deane, 2014; Chetty and Hanass-Hancock, 2016) thus placing unique demands on the health system. No studies were found exploring health system challenges affecting falls prevention services in primary care for PLWH from the perspective of physiotherapists. The aim of this study was to report on the health system challenges affecting physiotherapists’ management of falls in PLWH in four selected urban districts in four sub-Saharan countries. The findings from this study can inform policy-makers, researchers and primary health care workers including physiotherapists regarding opportunities for optimising provision of falls prevention services to PLWH.

## Methods

### Study design

This paper reports on a subset of interview data collected in a qualitative descriptive study that used an explorative phenomenological approach. An objective of the larger study was to explore physiotherapists’ perspectives and experiences regarding falls prevention for PLWH. Here we report on one theme that emerged from the study, namely ‘health system challenges’, following the consolidated criteria for reporting qualitative research (COREQ) (Tong, Sainsbury and Craig, 2007).

## Reflexive analysis

Qualitative research acknowledges that each researcher brings a unique perspective to the study. Reflexive analysis was done to improve confirmability of the study by the primary investigator (PI) acknowledging any influence or personal biases that may have affected the results of the study. The PI was the main interviewer although a second interviewer (QL or KB) was present to ensure consistency and coherence throughout the interview process.

All investigators were female physiotherapists and had experience in qualitative research. The interviewers revealed their identity and profession to prospective participants. Thus, it was expected that participants would be comfortable in using scientific or rehabilitation terminology in their responses.

### Study setting

The research was conducted in primary health care (PHC) facilities in the urban districts of capital cities (Gaborone, Cape Town Metropole, Harare and Lusaka) of four sub-Saharan African countries (Botswana, South Africa, Zimbabwe and Zambia). All four countries are classified as low-to-middle income countries (LMIC) (World Bank Group [US], 2019).

### Study population and sampling strategy

Physiotherapists currently practicing in public primary care facilities or hospitals and had at least two years’ experience in the primary care of PLWH were purposively sampled to participate in the study. A randomised list of PHC facilities from each selected district was created using the automated function in Microsoft Excel. Following these lists, the PHC facilities were telephoned to identify physiotherapists who met the inclusion criteria and were willing to participate in the study. Participant information booklets and consent forms were sent via e-mail to willing participants, in which the nature and purpose of the study was fully explained. Participants who returned their signed consent forms were further contacted to arrange for an interview within a month’s timeframe.

### Sample size

Purposive sampling methods place primary emphasis on data saturation (Patton, 2002). Participant recruitment continued until data saturation was achieved, that is, when only variations on themes already identified were being expressed without new ideas or themes emerging. It was proposed a priori that a maximum of five to six participants per country would be interviewed. This agrees with the recommended typical sample size for a phenomenological study (Creswell and Poth, 2017).

### Data collection

Data were collected via 20–30-minute-long individual telephonic interview conducted between December 2018 and July 2019. The PI conducted interviews at pre-appointed times that did not interrupt participants’ clinical practice. Both the interviewers and interviewees were seated in closed, quiet rooms free of disruptions. Non-participants were not allowed into the rooms of the interviewers nor interviewees whilst interviews were being conducted to ensure privacy and confidentiality. This endeavoured to allow interviewees to provide honest responses.

### Instrumentation

The interview schedule was informed by similar qualitative studies of rehabilitation specialists’ perceptions of falls prevention for the general geriatric population in primary care settings (Liddle *et al.*, 2018; Mackenzie and Clifford, 2018). Questions were open-ended and designed to specifically address physiotherapists’ care of adult (not necessarily geriatric) PLWH. Table 1 presents a sample of questions included in the interview schedule.

**Table 1.** Sample of questions included in interview schedule

*‘What are you currently doing for falls prevention in your delivery of care to PLWH?’*
*‘Which Clinical Practice Guidelines for falls prevention do you refer to?’*
*‘Which health care professionals do you refer your patients to for further falls risk prevention?’*
*‘Any recommendations regarding what physiotherapists need to do improve care of PLWH considering their potentially high risk of falls and reduced BMD?’*

The interviewer was prepared to digress from the interview schedule and ask probing questions to clarify key points raised. Leading or emotive questions that could have led interviewees into giving desired responses were avoided.

No pilot interview was conducted. However, the interviewer’s reflection after the initial three interviews led to the addition of questions that were applied to successive interviews after discussion with the co-researchers. New issues that arose during interviews and were relevant to the study objectives were also addressed in interviews with subsequent participants. No repeat interviews were conducted.

### Audio recordings

All interviews were recorded electronically after obtaining verbal consent from each participant. After allocating unique study IDs, electronic recordings were safely stored in a password protected file on the PI’s laptop.

### Note keeping

Interviewer notes were kept, noting key points and non-verbal cues such as tone of voice, reluctance or emphasis expressed by the interviewee during discussions. The PI kept a reflective diary to record personal reflections of values, interests and insights after each interview and decisions affecting the research process.

### Data processing

Electronic recordings of interview sessions were transcribed verbatim with the assistance of professional transcribers. The PI checked the accuracy of all transcripts and transcribed five interviews herself to develop this skill as well as allow for immersion in the data (Sutton and Austin, 2015). The co-researchers (KB, QL) checked six random transcriptions for accuracy in transcription.

Transcripts were returned to participants via e-mail for comments and/or corrections to ensure that transcribed accounts accurately reflected what they had said. Participants’ names were removed from the transcripts. Transcripts continued to be identified by the same unique IDs allocated to the audio recordings.

### Data analysis

Thematic content analysis with a deductive reasoning approach was applied (Patton, 2002). The analysis was informed by the WHO Health Systems Assessment Framework, which depicts health systems using six components: leadership/governance, financing, health workforce, medicine and technologies, information and service delivery (WHO, 2013).

Data analysis was an iterative and reflective process and involved constant moving back and forward between phases of data collection and analysis (Patton, 2002). The PI repeatedly read transcripts to immerse herself in the data. Theoretical and reflective thoughts that developed through immersion in the data were documented (Nowell *et al.*, 2017). The PI’s reflective diary and interviewers’ notes were also consulted during coding for information, which provided deeper insight and context to the recorded data (data triangulation). The transcripts were used to establish common themes into units of meaning or codes using a computer-assisted qualitative analysis software (Atlas.ti.8®). The co-researchers (KB, QL) independently hand-analysed three randomly-selected transcripts each. Codes were compared and discussed among the researchers and merged to create a codebook. Disagreements were resolved by discussion until consensus was achieved. Quotations which best represented each category were selected as supporting quotations. An audit trail was carried out by keeping an accurate record of all information and processes for other researchers to confirm.

## Results

### Participant characteristics

A total of 30 physiotherapists were invited to participate in this study. Seven physiotherapists declined participation due to fear of not being able to provide enough information on the topic, while one declined due to lack of time. Two physiotherapists who had given consent to participate could not be contacted on the scheduled appointments despite follow-up. Thus, a total of 21 interviews were conducted over a period of seven months. The PI did not have to recruit more participants because data saturation was achieved. Table 2 summarises the participants’ sociodemographic characteristics.

**Table 2.** Participant characteristics

Variable	Number (*n* = 21)	Percentage (%)
Gender		
Male	4	19
Female	17	81
Work setting		
General or Referral Hospital	13	62
District Hospital	6	29
Military Hospital	1	5
Primary Health Clinic	1	5
Cities (countries)		
Gaborone (Botswana)	6	29
Cape Town Metropole (South Africa)	4	19
Lusaka (Zambia)	6	29
Harare (Zimbabwe)	5	24
Highest professional qualification		
BSc. Physiotherapy	17	81
MSc. Physiotherapy	4	19
Age in years (mean ± SD; IQR)	33.76 ± 4.22; 26–43	
Years of professional experience (mean ± SD; IQR)	9.67 ± 4.22; 3–19	
Years of caring for PLWH (mean ± SD; IQR)	8.90 ± 4.34; 3–19	

### Main findings: Participant-derived theme and categories

A major theme that arose from the larger study’s data was ‘health system deficiencies’. Participants’ accounts revealed factors that may indicate deficiencies in the quality, efficiency and equity of the health care system that adversely affected their management of falls risk and bone demineralisation in PLWH. The categories (mapped under each building block of the WHO Health System Framework) are presented below (Table 3). Selected supporting verbatim quotes are also presented.

**Table 3.** Summary of theme and categories

Theme	WHO Building block	Category
Health system challenges	Leadership/governance	Lack of policies and clinical practice guidelines
	Health care financing	Shortage/Inaccessible falls prevention services
	Health workforce	Inadequate human resource
		Physiotherapists not adequately equipped in falls prevention
	Medical technology	Inaccessible/no facilities for bone mineral density measurement
	Information and research	Inefficient data capturing systems
		Lack of evidence regarding falls among PLWH
	Service delivery	Unclear physiotherapy role descriptions
		Inefficient referral system

### Leadership/governance

#### Lack of policies and clinical practice guidelines

None of the countries had independent policy on physiotherapy-specific care of PLWH, including falls prevention in PLWH. This is exemplified in the following quote:

*‘…there is no specific policy to say physiotherapy, but there is a general guideline on the management of HIV. But not something which is strictly…referring to my profession alone, no.’* Participant 12, Zambia, general hospital.

Particularly in Botswana, where some hospitals are currently undergoing accreditation, some effort was being made on development and implementation of falls prevention policies for all hospitalised patients (which inherently include PLWH). Physiotherapy departments were expected to adapt these guidelines, but very few reported having departmental policies on generic falls prevention. As such, policies guiding falls prevention for community-dwelling PLWH were non-existent.

### Health care financing

#### Shortage of/inaccessible falls prevention services

Physiotherapists reported that fall prevention services were not available to PLWH at primary care level, due to the shortage of assistive devices in most facilities. Some participants from all the countries indicated that patients were unable to access required falls prevention services because they could not afford it.

*‘…lack of access to appliances like wheelchairs, we have serious shortage of wheelchairs and then the other thing is like crutches, walking aids.’* Participant 2, Botswana, district hospital.

*‘Some people will tell you we don’t have money to buy assistive devices, we can’t afford to buy crutches or walking frames.’* Participant 19, Zimbabwe, referral hospital.

### Health workforce

#### Inadequate human resource

A disproportionate number of physiotherapists in our sample population was employed in the primary care of PLWH. One participant pointed out the lack of clear-cut role descriptions for involvement of physiotherapists in the care of PLWH, often resulting in physiotherapists being side-lined from the care of PLWH.

*‘What we find is the role of physiotherapy within the whole spectrum of HIV and patient care is quite limited particularly by the authorities… or you find there are not many physiotherapists that are employed in that sector, and yet I think there is quite a big role to play.’* Participant 17, Zimbabwe, referral hospital.

There was also a lack of other health professionals at primary care level. Therefore, some of the specialist services were not available at their hospitals or clinics for physiotherapists to refer patients for further falls prevention services.

#### Physiotherapists not adequately equipped in falls prevention

Most physiotherapists reported inadequate knowledge and skills in falls prevention for PLWH, citing inadequate prelicensure training. Physiotherapists from Botswana presented a unique scenario, in which the local non-existence of physiotherapy training schools meant they had to train in high-income countries, where HIV/AIDS is not a priority. These physiotherapists felt incompetent in caring for PLWH upon returning to Botswana.

*‘We did one page of HIV training. I studied at Australia; they didn’t tell much on HIV. It was my first time seeing it when I started working in Botswana, so I just learnt through experience and asking colleagues.’* Participant 4, Botswana, district hospital.

Unequal opportunities for professional training were reported. Most health facilities prioritised the training of medical doctors and pharmacists in the provision of care to PLWH while limited opportunities existed for physiotherapists.

*‘… maybe that is where this overlooking thing comes up because really when they do their HIV workshop, they don’t incorporate rehab staff or the physios.’* Participant 21, Zimbabwe, referral hospital.

### Medical technology

#### Inaccessible/no facilities for BMD measurement

An absence of dual energy X-ray absorptiometry (DXA), considered the gold standard for measurement of BMD, was reported as a deterrent to physiotherapists’ ability to routinely assess the risk of bone demineralisation in their patients. Most reported using X-rays as the main diagnostic tool for osteoporosis, with limited use of computer topography (CT) due to its high cost.

### Information and research

#### Inefficient data capturing systems

Participants noted that inefficient capturing of statistics prevented physiotherapists from being able to notice trends indicating fall risk among PLWH.

*‘…I think maybe it’s also because when I treat them, I haven’t really tallied or taken statistics to compare those with HIV to those without really. So, I can’t really say I have seen more of the other than the other.’* Participant 1, Botswana, military hospital.

#### Lack of evidence regarding falls among PLWH

Participants further indicated poor coverage of information regarding care of PLWH, including falls prevention, in their undergraduate curricula. In these curricula, falls prevention was considered in the area of gerontology and not linked to HIV.

*‘I don’t recall very clearly going into the details of it…I have seen there has been quite a few articles on the research of HIV management in the physiotherapy field, but I cannot recall touching on it so in-depth as an undergraduate.’* Participant 9, South Africa, district hospital.

### Service delivery

#### Unclear physiotherapy role descriptions

Several participants highlighted how other health professionals were unaware of the roles that physiotherapists played in falls prevention or primary care of PLWH. As a result, physiotherapists were often excluded from HIV policy workshops while PLWH were not referred adequately for physiotherapy-led falls prevention.

*‘Apparently here in Zimbabwe, maybe that is where this overlooking thing comes up because really when they do their HIV workshop, they don’t incorporate rehab staff or the physios.* Participant 21, Zimbabwe, referral hospital.

*‘I don’t think we would ever get referral from a doctor saying that please assist this patient …because he is high risk of falling. That very rarely happens, I am going to go with one percent of referrals.’* Participant 7, South Africa, district hospital.

#### Inefficient referral system

Concerns were raised regarding the referral system between members of the multidisciplinary team (MDT) that could contribute to reducing falls risk in PLWH. The doctors remained the gatekeepers to the patients. The nurses could not refer to other members of the MDT, even though they were better positioned to identify patients at risk of falls.

*‘…we don’t screen our patients in the ward because then literally every single patient in the hospital would get some form of physiotherapy and then we are looking at four hundred patients getting physio on a daily basis which we do not have the capacity to do, so unfortunately we do work on a purely doctor referral basis.’* Participant 7, South Africa, district hospital.

*‘In our hospital the nurses are not allowed to refer to the allied health…but I am sure that the nurses do pick up the concern…they can verbally raise the concern with us and then we will advise them to speak to the doctor who will then refer the patients to us.’* Participant 9, South Africa, district hospital.

#### Lack of community re-integration programmes

Another deficiency in the health care delivery system was failure to ensure community reintegration of patients and ensuring social and financial support. The doctors reportedly discharged patients before they had achieved sufficient mobility status and were at high risk of falls.

*‘And as soon as the person is cleared medically and stable, they should get discharged whether they are able to walk or not. More or less that is what the hospital does.’* Participant 19, Zimbabwe, referral hospital.

## Discussion

To our knowledge, this is the first study to describe health system challenges as a barrier to integrating falls prevention into the primary care of PLWH, as perceived by physiotherapists from sub-Saharan African regions. It was clear that the provision of falls prevention services was suboptimal in all four investigated regions. This was partly attributed to gaps in the health system related to all six building blocks of the WHO health systems framework, namely leadership/governance, health care financing, health workforce, medical technology, information and research and service delivery. Mapping our findings against the established building blocks of a strong health system enabled us to propose potential strategies to implement falls prevention services targeted at PLWH into primary health care settings.

### The need for quality clinical practice guidelines

Barriers to the development and implementation of clinical practice guidelines (CPG) by the allied health workforce in LMIC primary care settings have been identified (Dizon *et al.*, 2017). These include financial constraints and health policy priorities. To date, HIV policy-makers have placed more focus on the medical and pharmacological management of HIV to curb new transmissions and reduce HIV-related morbidity and mortality (Cobbing, Hanass-Hancock and Deane, 2014). More guidelines are needed to guide improvement of QoL for PLWH who are now living longer. A lack of CPG can result in mismanagement, undertreatment or overtreatment of patients which may translate into unnecessary financial costs or negative impacts on their health outcomes (Kredo *et al.*, 2016). Phelan *et al.* (2015) developed guidelines on assessment and management of fall risk in older adults of the general population in primary care settings. Such guideline may be implemented for all fall-prone patients and simultaneously make physiotherapists aware of the potential risk of falls among PLWH. Implementation of these guidelines may guide physiotherapists in making better clinical decisions informed by best evidence resulting in improved health outcomes for PLWH (Culleton, 2015).

Despite this gap, our results also highlighted the potential positive outcomes of using the hospital accreditation system as a vehicle of CPG development and implementation. For example, physiotherapists reported that quality improvement initiatives in Botswana fostered the development of quality CPG for generic falls prevention by bench-marking against recognised standards of practice (Kredo *et al.*, 2016). Considering that most evidence on falls prevention currently hails from HICs, the guidelines need to be contextualised to LMICs where there are variable local resources, budgetary constraints and different patient needs (Dizon *et al.*, 2017). For CPG to be patient-centred, PLWH will need to be involved as important stakeholders in the development of these guidelines and active participants for successful implementation.

### Health care finance

Participants voiced their concern regarding PLWH not being able to access falls prevention services due to cost or unavailability. The economic costs of falls prevention services associated with the purchase of assistive devices, attendance fees and transport costs are commonly-reported barriers to implementing falls prevention services – cited in over two-thirds of studies included in a systematic review (Child *et al.*, 2012). Although much health care funding has been dedicated to HIV care in LMIC, (GBD 2017 Health Financing Collaborator Network, 2018) significant proportions have been allocated to virological control of HIV through ART scale-up, voluntary male circumcision, prevention of mother-to-child transmission, universal testing and immediate treatment programmes (GBD 2017 HIV Collaborators, 2019). Not disregarding the need for (and success of) such initiatives, a shift in focus may be required to improve the QoL of PLWH along with the extended lifespans. An investment case for concerned stakeholders regarding falls prevention in PLWH may be warranted to prioritise funding in this area.

### The need for more information databases and research on falls in PLWH

Physiotherapists highlighted poor capturing of statistical data on falls occurring in PLWH as another gap in the health system as a barrier to falls prevention in PLWH. Their failure to document falls may be linked to their lack of routinely assessing for falls in PLWH or to patients failing to report fall incidences (Phelan *et al.*, 2015). It is well-reported that data in patient health records that are collected during routine health care activities facilitate contextual clinical research (Cowie *et al.*, 2017). Regarding the prevalence of falls in PLWH, most studies have been conducted in high-income countries, and mostly in middle-aged and older PLWH (Erlandson *et al.*, 2016, 2019; Sharma *et al.*, 2016, 2018). Feasibly, falls prevalence could be higher in SSA where more robust strains of the HIV-1 Claude C virus exist and may have greater CNS neurotoxicity (Mahadevan *et al.*, 2007; Rademeyer *et al.*, 2016). Furthermore, the risk profile of PLWH in poorly-resourced settings with sub-optimal health systems may also be different, due to coexisting comorbidities, poor ART adherence and malnutrition (Pathai *et al.*, 2013). Prevalence data would enable the definition of the burden of falls among PLWH in SSA and contribute towards an understanding of the need for falls prevention services. Such data would aid policy-makers in planning financial and resource needs accordingly.

The information and research on falls in PLWH may also be important in training physiotherapists and other health care professionals. The physiotherapists in our study perceived their prelicensure training and continuous professional development on falls prevention for PLWH as inadequate. Thus, most physiotherapists’ knowledge on falls prevention was based on what they were taught in another context, such as gerontology. An audit of the physiotherapy undergraduate curricula of eight South African academic institutions by Myezwa *et al.* (2008) revealed scanty HIV-related content. They recommended revision of the undergraduate curriculum to include aspects of care of PLWH and for rehabilitation professionals working with PLWH to keep up to date with current evidence. With added research evidence, opportunities for continuous professional development may be created for physiotherapists, helping to bridge the gap especially for physiotherapists trained in HIC where little emphasis was put on HIV care. This will improve the health care workforce’s competency in delivering falls prevention services to PLWH.

### The need for promotion of physiotherapists’ role in primary care of PLWH

This study highlighted additional health system challenges stemming from other health care professionals’ poor understanding of the scope of physiotherapy practice in the primary care of PLWH, including falls prevention. This resulted in a disjointed pathway of care that negatively impacted health outcomes for the patients.

Lack of other health professionals’ knowledge about physiotherapists’ role in HIV care resulted in the profession being misunderstood or undervalued (Cobbing *et al.*, 2013). It could be the reason why physiotherapists and other rehabilitation specialists were often side-lined from HIV workshops and policy dialogues as was found in this study. Similar concerns were reported by rehabilitation professionals in Kenya and Zambia (Nixon *et al.*, 2016); outlining some rehabilitation roles in HIV care that were overlooked: encouraging drug adherence, identifying impairments resulting from HIV infection or ART, offering psychosocial counselling, promoting self-management strategies, promoting mental health and advocating for rights of PLWH. An understanding of professional roles within teams can be acquired through interprofessional collaboration during undergraduate education (Berman, 2013). Practicing clinicians may be educated by means of courses, workshops, team meetings, grand rounds and research publications (Tadyanemhandu *et al.*, 2016). In collaboration with other rehabilitation professions, physiotherapists should imprint their hallmark in primary HIV care (Cobbing *et al.*, 2013). This may lead to their inclusion in HIV policy and programme development. Issues such as fall and fracture prevention may then be integrated into priority programmes and HIV guidelines.

Physicians not knowing what physiotherapists do for falls prevention may have contributed to physicians inadequately referring PLWH to physiotherapy as mentioned by a few physiotherapists in this study. An inadequate referral pathway was similarly reported in another South African study, (Maddocks *et al.*, 2018) where doctors explained that sometimes they did not refer PLWH for physiotherapy because they doubted the effectiveness of physiotherapy interventions in the management of HIV-related conditions. This may have resulted from the undergraduate medical curriculum having been curative-centric (Maddocks *et al.*, 2018). Hence, their treatment focus would be medical, affording PLWH little opportunity for being referred to rehabilitation services – when such services may have been needed (Chetty and Hanass-Hancock, 2016). Increasing physicians’ awareness of the role of physiotherapy in fall and fracture prevention may facilitate effective referral practice.

An Irish study (Mackenzie and Clifford, 2018) found that while rehabilitation professionals rarely received specific referrals for falls prevention from general practitioners, nurses did refer adequately. However, in the present study, participants reported that nurses were not allowed to refer to rehabilitation professionals despite being responsible for screening and identifying fall prone patients. This could be due to traditional hierarchical structures, or a fear that nurses will refer inappropriately (Mian, Koren and Rukholm, 2012). Moving away from physician-centric models of PHC to team models, (Golden and Miller, 2014) and allowing PHC nurses as first-line practitioners to directly refer patients to other HIV care providers, may result in increased and timely referral of PLWH for falls and fracture prevention.

A limited understanding of physiotherapists’ role in PHC may have been demonstrated by the smaller proportion of physiotherapists involved in this study who were employed in PHC settings. In fact, only one of the physiotherapists was employed at an outpatient HIV clinic while less than a third worked in primary district hospitals. Cobbing *et al.* (2013) highlighted the shortage of physiotherapy posts in many South African government institutions, despite many physiotherapists being unemployed and despite the demand for rehabilitation, mainly because the profession is undervalued. The same concerns have previously been raised by rehabilitation providers in Kenya and Zambia, who explained how their preventative role was disregarded; rehabilitation often being viewed as a ‘dumping ground’ after other treatment had failed (Nixon *et al.*, 2016). Governments may need to build capacity and availability of physiotherapists in primary care sectors and HIV outpatient settings.

Physiotherapists’ role in PHC seems to be more appreciated in high-income countries. Deboer *et al*. (2019) reported positively on health care professionals’ awareness of the preventative role of physiotherapy in an interprofessional outpatient HIV care setting, including falls prevention and prevention of secondary complications and pain. Besides provision of physical treatment, physiotherapists’ contributions to psychosocial aspects of health of PLWH were recognised. Suggested methods that physiotherapists can use promote their role in PHC include making educational visits to HIV out-patient clinics, as well as canvassing on social media platforms (Deboer *et al.*, 2019). If physiotherapists are engaged at primary level of HIV care, there may be improved focus on primary fall and fracture preventions.

### Need for patient-centred approach to fall prevention

**Figure 1.**
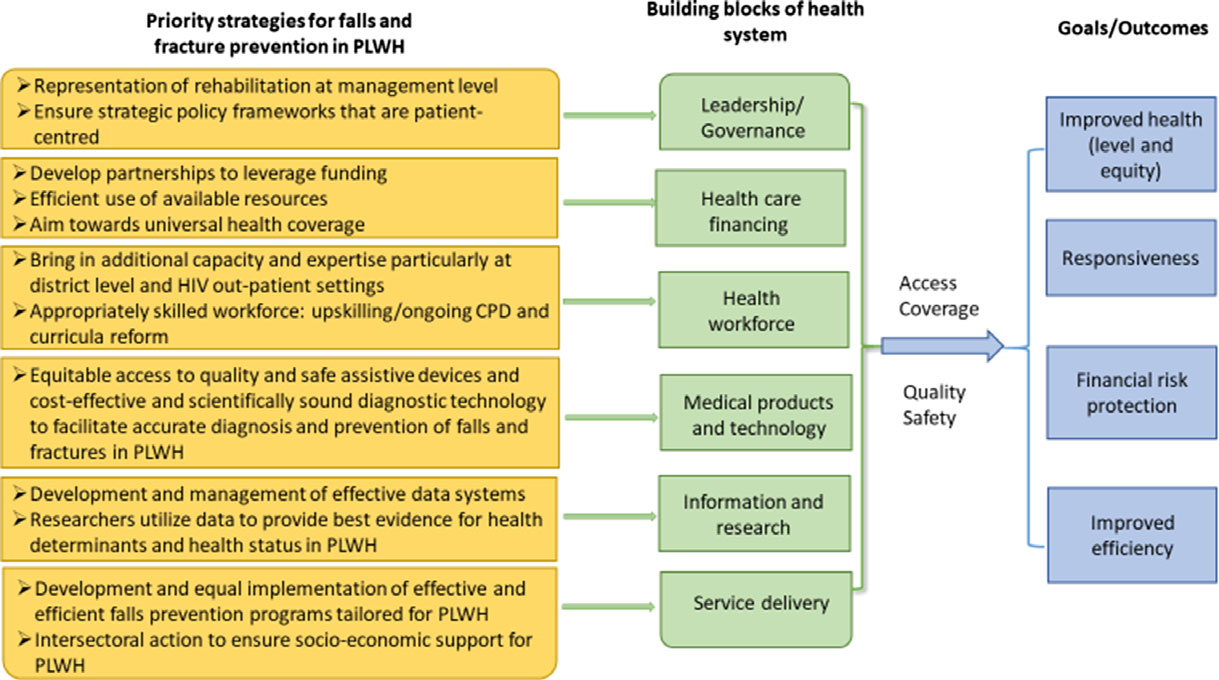
Proposed strategies derived from this study that can strengthen health systems (adapted to the WHO Health Systems Framework).

Another gap in the health system affecting falls prevention for PLWH was the lack of some elements of patient-centred care. Some participants indicated poor socio-economic support from PLWH’s families as a hindrance to successful implementation of falls prevention services. A similar lack of caregiver support for patients’ fall management were raised in a Malaysian study, (Loganathan *et al.*, 2015) and recommended education of caregivers on the importance of fall prevention and encourage them to report falls to health care providers. Additionally, engaging PLWH as active participants in individualised treatment may ensure successful implementation of falls prevention strategies (Epstein and Street, 2011). Intersectoral action within health systems will also ensure that PLWH are safely reintegrated into their communities socially and economically (Ndumbe-Eyoh and Moffatt, 2013). Figure 1 summarises the strategies identified from this study to address health system challenges in falls prevention in primary care of PLWH.

### Limitations of the study

Purposive sampling was used to gain perspectives of physiotherapists working in primary care of PLWH. However, physiotherapists in Zimbabwe and Zambia were mostly employed in secondary and tertiary hospitals, while a disproportionate number of physiotherapists in South Africa worked in the private sector. Participants were restricted to the public sector but included the input of physiotherapists employed at general and referral hospitals because they were still the first line of contact for rehabilitation and would be the same physiotherapists who would need to implement falls prevention programs in the community perhaps by means of outreach visits to primary hospitals and HIV clinics. By doing so, the study gained additional information with regards to falls that occur in hospitalised PLWH. Perhaps maximum variation sampling would have better suited this population and allowed the researcher to assess differences in patterns of experience from physiotherapists engaging with PLWH at different levels of care.

## Conclusion

A strong patient-centred health system is essential for the management of the health needs of the community including PLWH. Findings from this study indicate that physiotherapists face various challenges from deficiencies within the health system when it comes to falls prevention for PLHW, including fragmented service delivery, shortage of competent human resources and budgetary constraints. Physiotherapists play a crucial role in re-integrating PLWH into communities and need to liaise with other health professionals to ensure patient-centred continuum of care for PLWH and effectively improve their QoL.
